# Measuring inter-individual differences in stress sensitivity during MR-guided prostate biopsy

**DOI:** 10.1038/s41598-021-82199-z

**Published:** 2021-01-28

**Authors:** Nils Kohn, Jan Heidkamp, Guillén Fernández, Jurgen Fütterer, Indira Tendolkar

**Affiliations:** 1 Donders Institute for Brain Cognition and Behavior, Department of Cognitive Neuroscience, Radboud University Medical Center, Nijmegen, The Netherlands; 2 Department of Medical Imaging, Radboud University Medical Center, Nijmegen, The Netherlands; 3 Donders Institute for Brain Cognition and Behavior, Department of Psychiatry, Radboud University Medical Center, Nijmegen, The Netherlands

**Keywords:** Cognitive ageing, Cognitive neuroscience, Emotion, Stress and resilience

## Abstract

People often experience high level of distress during invasive interventions, which may exceed their coping abilities. This may be in particular evident when confronted with the suspicion of cancer. Taking the example of prostate biopsy sampling, we aimed at investigating the impact of an MRI guided prostate biopsy on the acute stress response and its mechanistic basis. We recruited 20 men with a clinical suspicion of prostate cancer. Immediately before an MRI guided biopsy procedure, we conducted fMRI in the same scanner to assess resting-state brain connectivity. Physiological and hormonal stress measures were taken during the procedure and associated with questionnaires, hair cortisol levels and brain measures to elucidate mechanistic factors for elevated stress. As expected, patients reported a stress-related change in affect. Decreased positive affect was associated with higher hair but not saliva cortisol concentration. Stronger use of maladaptive emotion regulation techniques, elevated depression scores and higher within-salience-network connectivity was associated with stronger increase in negative affect and/or decrease of positive affect during the procedure. While being limited in its generalization due to age, sample size and gender, our proof of concept study demonstrates the utility of real-life stressors and large-scale brain network measures in stress regulation research with potential impact in clinical practice.

## Introduction

Life expectancy is increasing worldwide. Unfortunately, these additional years are not always spent in good health. Population aging leads to an increase in different forms of cancer, which need to be diagnosed rapidly and precisely. To facilitate diagnosis, medical disciplines like radiology have tried to develop minimally-invasive diagnostic tools. Despite these technical advantages, in particular elderly patients often experience a high level of distress during an intervention which may exceed their coping mechanisms^1^. Flory and Lang have shown that patients awaiting diagnostic procedures at the radiology department commonly experience high stress levels with perceived impact on daily life^2^. With respect to diagnostics of potential malignancies, it is important to take into account that uncertainty about the diagnosis can elicit additional distress and not only the mere anticipation of a risky invasive therapeutic procedure^2^. Moreover, stress reactivity may depend on the individual level of emotion regulation capacity.

Effects of acute stress have most often been investigated in standardized laboratory settings in groups of healthy young students. Few studies included elderly groups or focused on possible age differences in effects of acute stress. Findings on the relation of age and emotion processing and experience are mixed. Some studies find better emotion regulation abilities in older adults when acutely stressed, others found a reduction of positive emotions or no age differences^3^. The mechanistic underpinnings of putative age related changes in emotion regulation are unclear^4^. In addition, while age-related neural changes in learning and memory have been studied extensively, relatively few studies have investigated the neural correlates of emotional aging. We could show in a recent study comparing healthy young and elderly men that healthy older adults display elevated activity in brain regions involved in emotion processing during a mild stressor induction procedure^5^. These findings suggest that increased reactivity in emotion processing brain regions during acute stress in an otherwise healthy older individual may constitute one mechanism by which leads into heightened vulnerability for affective disorders. This association may explain why acute distress in the elderly can lead to longer lasting emotional problems. However, studies investigating acute real-life stressors with potential impact on clinical practice in an elderly population are lacking.

Stress reactivity is associated with dynamic changes in functional brain networks, measured as patterns of synchronized activity across a set of distributed brain regions^6^. Three networks in particular are involved in task and rest related brain function, i.e. the salience network (SAL), the default mode network (DMN) and the central-executive network^7^. In health, the stress response comprises dynamic shifts in these three networks, that facilitate adaptive coping with the stressor^8,9^ and explain concurrent cognitive performance^10^. In particular, acute stress is accompanied by an upregulation of the salience network and a relative decrease in connectivity of the executive control network. It is yet unclear whether and how this acute stress reaction unfolds in elderly individuals and also little knowledge exists whether these dynamics are similar in a real life stressor.

In the light of the aforementioned background, we take the example of a minimally invasive intervention as a real-life stressor that is applied in elderly. We assume its stress inducting effects to be generalizable to the general elderly population. We recruited 20 men with a clinical suspicion of prostate cancer. We used an innovative technical set-up of MRI-guided prostate biopsy. Directly before an MRI guided biopsy procedure, we conducted fMRI to assess resting-state brain connectivity. Physiological and hormonal stress measures were taken during the procedure and associated with questionnaires, hair cortisol levels, and brain measures to elucidate mechanistic factors for elevated stress. We aimed at investigating the interaction of neural and cognitive patterns concerning stress and resilience in these otherwise healthy elderly males. Prostate cancer is the most commonly diagnosed malignancy in men in developed countries. Prostate biopsy in many individuals leads to elevated anxiety that is not only related to the potential positive outcome of the biopsy but also potential side effects. This renders the intervention highly stressful. Even in patients who received benign results, adverse psychological, psycho-social, and medical outcomes have been observed up until 1 year after the intervention^11^ thus leading to psychological, socio-behavioral, and medical care implications. Despite an optimization in prostate cancer screening with multi-parametric magnetic resonance imaging (MRI), MRI in itself may be a potentially stressful experience^12^, which may add to the stress burden of biopsy and cancer threat.

## Results

**Figure 1:**
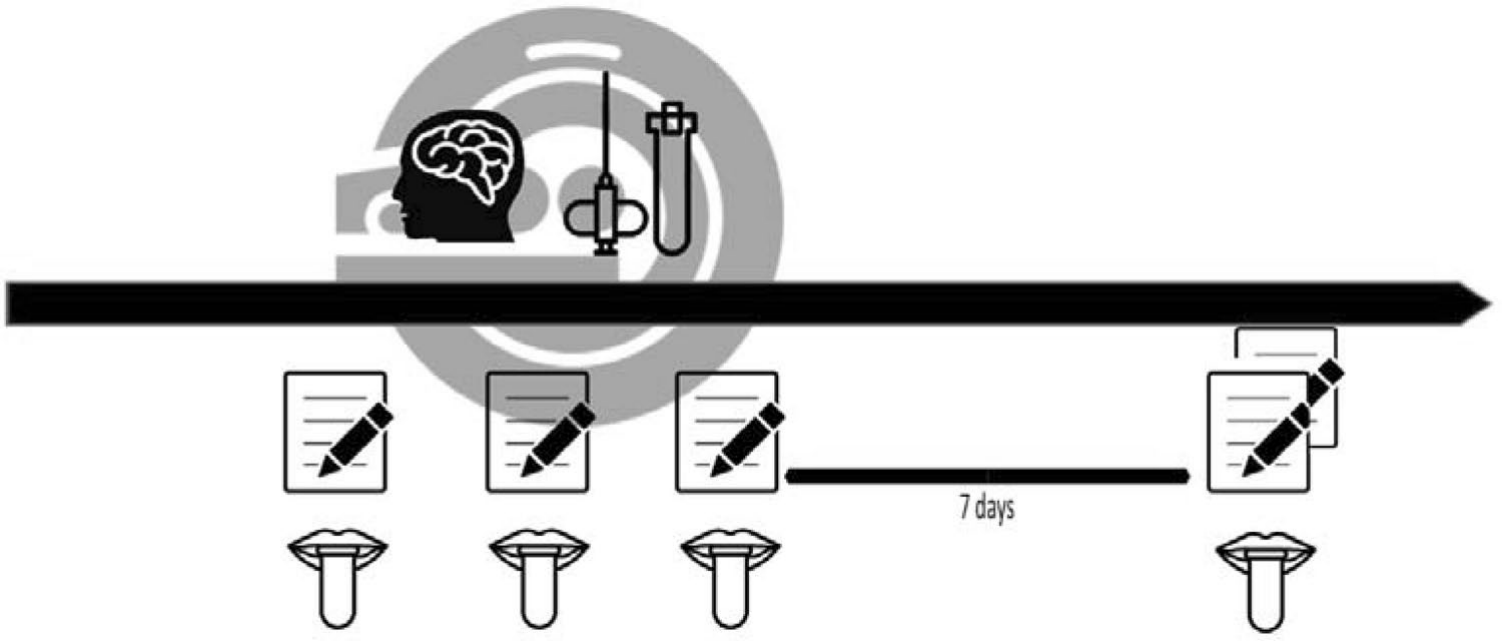
Schematic overview of the study design. The lower level timescale describes the days before and after biopsy, the upper figure describes the measurements taken during the biopsy procedure.

In order to explore the peri-interventional stress during prostate biopsy we investigated a physiological stress response (salivary cortisol) and a subjective stress response (positive and negative self-ratings of affect). We furthermore included possible mediating factors such as physiological indicators of long term stress (hair cortisol), large-scale brain network connectivity related to stress and emotion regulation strategies. Brain data and hair cortisol was acquired just prior to the start of the MR-guided prostate biopsy. We took subjective affective self-assessments and salivary cortisol levels before the biopsy procedure, prior to the brain scan and at the end of the entire procedure (Fig. 1).

Twenty men between 50 and 70 years of age (mean age: 64.9 ± 5.3 years) with a high suspicion of prostate cancer participated in the study. Below we describe the influence of stress during MR-guided prostate biopsy on behavioral, physiological and brain measures and their predictive factors in more detail.

### Affect ratings during MR-guided biopsy procedure

**Figure 2:**
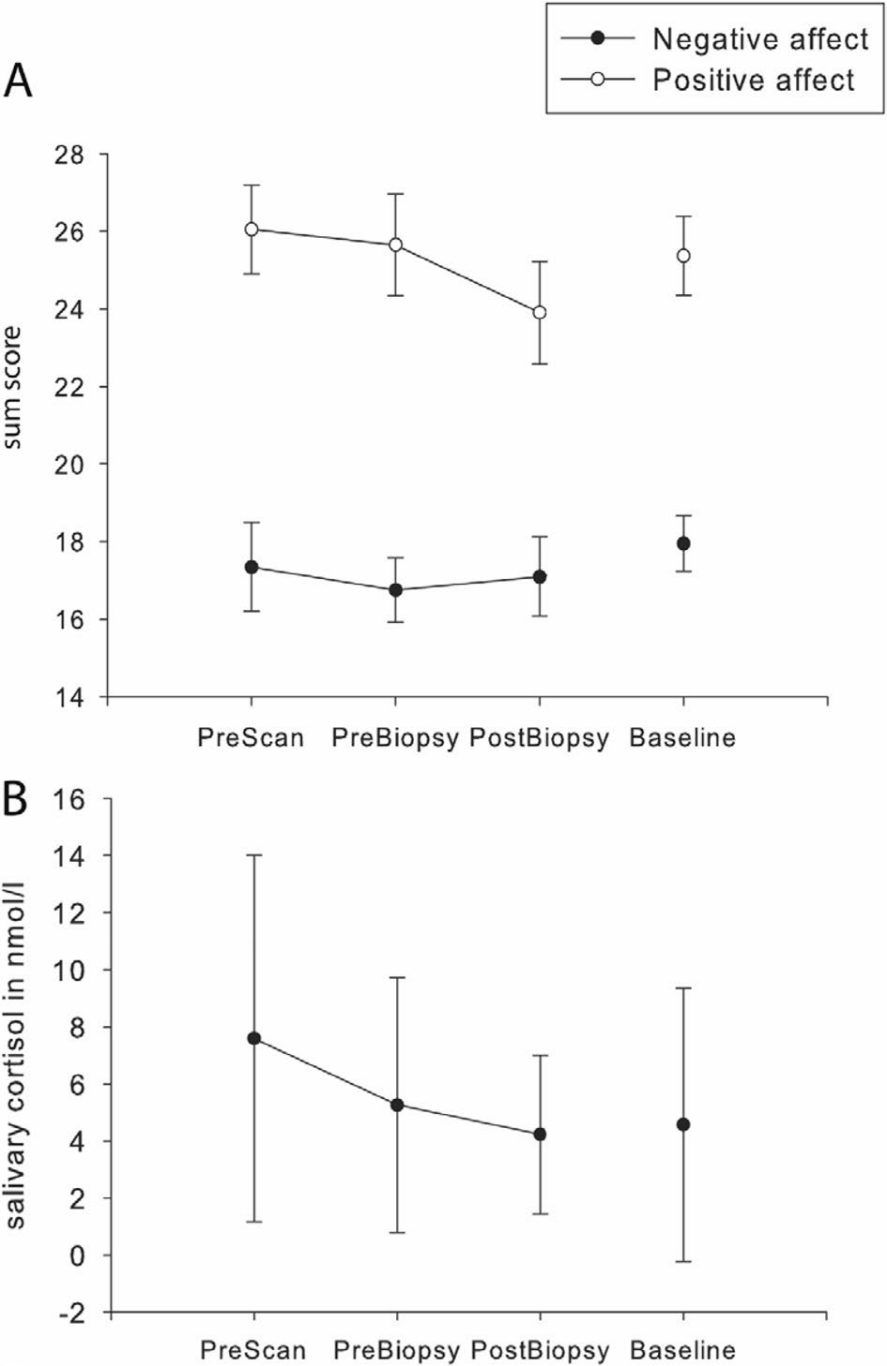
(**A**) Mean PANAS scores for positive and negative subjective ratings of current affective state at different stages of the procedures. Error bars indicate one standard deviation. (**B**) Mean salivary cortisol values measured at time-points of the biopsy. Error bars indicate one standard deviation.

The descriptive values of the positive and negative affective state and the salivary cortisol are summarized in Fig. 2A for subjective affect ratings and in Fig. 2B for salivary cortisol values. We summarized descriptive values of sum scores of the questionnaires in Table 1.

**Table 1:** Mean values, standard deviations and sample size for sum scores of the questionnaires.

	N	Mean	Std. deviation
Hair cortisol pmol/mg	16	0.03	0.02
DASS_Depression	19	2.53	2.32
DASS_Anxiety	19	0.79	1.08
DASS_Stress	19	3.68	2.43
BDI	19	6.63	6.906
ERQ_Reapraisal	19	24.26	6.89
ERQ_Suppression	19	15.63	3.96
CISS_Task	19	58.47	9.65
CISS_Emotion	19	30.89	7.99
CISS_Distraction	19	15.79	6.592
CISS_Company	19	14.32	4.27
CISS_Avoidance	19	38.63	11.48

DASS21: A-anxiety (norm 1.75[3.13]), D-depression (norm 2.8[4.1]), S-distress (norm 4.7[3.61])^13^.

BDI: cut-off 13 for mild depression.

CISS-21: T—task oriented coping, E—emotion oriented coping, A—avoidance oriented coping.

ERQ: Cognitive reappraisal (norm 29[6.68]), Expressive suppression facet (norm 15.97[5.16])^14^.

The normative value is given in mean and SD in brackets.

We tested for changes during biopsy in saliva cortisol and subjective measures of affect while including brain state, hair cortisol and questionnaire data as covariates.

The repeated measures ANCOVA for cortisol did not reveal any significant main effect of or interactions with factors or covariates (all p > 0.1). Due to missing data on the cortisol sampling, we could only include 13 patients in this analysis.

In the analyses of subjective affective state, four patients had missing data on one of the questionnaires hair cortisol and where excluded. Thus, the analyses were conducted on 16 subjects. The repeated measures ANCOVA for subjective affect yielded a significant effect of the repeated measurement during the biopsy (F_1.826,19.467_ = 6.8; p = 0.007; partial η^2^ = 0.382). This effect was driven by a decrease in positive PANAS ratings and an increase in negative PANAS ratings during the biopsy procedure. The interaction between hair cortisol and repeated measurements during the biopsy was significant (F_1.826,19.467_ = 5.694; p = 0.026; partial η^2^ = 0.292). A higher concentration in hair cortisol was associated with a stronger drop in (most pronounced) positive PANAS ratings over the course of the biopsy (t-value dropped from 0.11 at first measurement to − 2.24 at last). The effects of time during biopsy were more pronounced in positive affect compared to negative affect, although the interaction between repeated measurements during the biopsy and valence was not significant, but below p = 0.1 (F_1.771,19.467_ = 3.16; p = 0.07; partial η^2^ = 0.223). Similar trend-like effects were observed in the interaction between repeated measures during the biopsy and connectivity strength of the DMN (F_1.826,119.467_ = 2.792; p = 0.089; partial η^2^ = 0.202). Higher within-network DMN connectivity strength was associated with less change in positive and negative affect throughout the procedure. All others main effects and interaction were not significant (p > 0.1).

### Large-scale brain network response in anticipation of prostate biopsy

**Figure 3:**
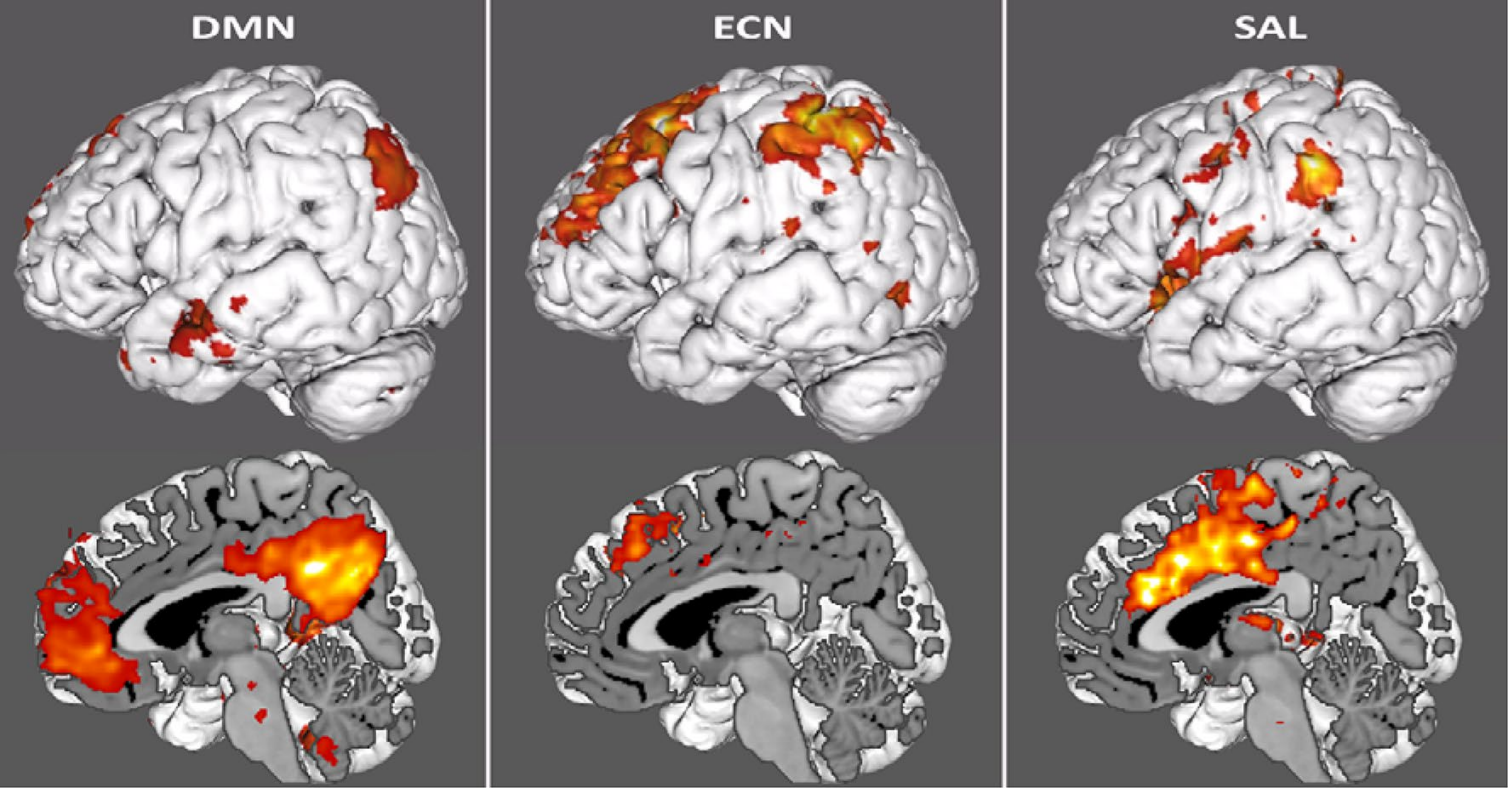
We display the group results for the three networks. Network connectivity is arbitrarily thresholded at z > 5 for display purposes.

We analyzed functional connectivity of all patients in anticipation of MR-guided prostate biopsy. We used templates of the three large-scale brain networks (SAL, EN & DMN) that have been shown to be involved in the acute stress response (see videos in supplementary material^9,10,15,16^; masks available in Neurovault: https://neurovault.org/collections/MFDMBANR/). We used these masks to estimate the average within network functional connectivity strength during the resting state measurement right before the prostate biopsy (Fig. 3; videos of these results in supplementary material; all results data in Neurovault: https://neurovault.org/collections/MFDMBANR/).

As a measure of upregulated SAL and downregulated ECN we estimated also the between network connectivity. We chose to describe the extend of *between* network connectivity by the percentage of connected voxels *within* a second network exceeding a z > 5 to a first network (for example, in between network connectivity of ECN to SAL, we report the percentage of voxels within a SAL mask that have a z > 5). We also display these for DMN as previous research has shown that social stress leads to an elevated connectivity between DMN and SAL hubs^15^. For the DMN, 93.4% of voxels in the template mask of the DMN were above z of 5, which signifies that the DMN was strongly and coherently connected in the group. 4.2% of voxels in the salience network template were connected to DMN and 1.3% of ECN template voxels to DMN. For the salience network, 71.2% of voxels from the salience template demonstrated a connectivity above z > 5. 8.9% of voxels within the ECN template were connected to the salience network and 0.06% of DMN template voxels. For executive control network, 67.2% of voxels in the ECN template mask were connected. ECN was connected to 1.4% of SAL voxels and 0.9% of DMN voxels.

### Association between subjective and physiological stress

We aimed to explore the between subject association of the physiological and affective stress response by questionnaires and brain data in an explorative step-wise regression. The regression of physiological stress reactivity (the difference between first and last saliva sample) no predictor was included in the initial step. Thus, we could not predict changes in cortisol levels.

To predict changes in positive affect yielded in the first step the DASS depression score as predictor and in the second step the ERQ suppression score. In total, the final solution resulted in an adjusted R^2^ = 0.696. The DASS depression score was positively associated with changes in positive affect (Beta = 0.595, p = 0.002), which indicates that higher depression scores are associated to a stronger drop in positive affect. The ERQ suppression score was negatively associated to change in positive affect (Beta = − 0.592, p = 0.002), indicating high tendency toward suppression is associated to a smaller drop in positive affect.

Changes of negative affect over the course of the biopsy was associated with emotional suppression as indexed by the ERQ and with SAL network connectivity strength. In total the final solution resulted in an adjusted R^2^ = 0.72. Both predictors were negatively associated (ERQ suppression: Beta = − 0.926; p < 0.001; Salience connectivity Beta = − 0.519; p = 0.004), indicating that the stronger use of suppression as emotion regulation tendency and higher within network connectivity strength of the SAL is associated with stronger increase in negative affect over the course of the biopsy.

## Discussion

This is to the best of our knowledge the first study that tried to assess the acute real life stress of an invasive diagnostic intervention like prostate biopsy in an otherwise healthy sample of elderly men. Given the complexity to integrate experimental brain imaging in a standardized minimally invasive diagnostic procedure, we only measured 20 male subjects and focused on signs of acute distress at the behavioral, physiological and brain level. The clinical questionnaires such as the DASS- 21 and the BDI showed that our subjects had no signs of clinical affective symptoms prior to the procedure. However and relevant with respect to the study question at hand, they revealed stress reaction as a function of the biopsy procedure. Future real-life stressor studies in the field of interventions should aim at extending the research question by relating individual stress reaction to pre-procedure assessment. In line with other studies measuring acute stress in elderly men^5^ we did not find a change in cortisol saliva concentration, while the PANAS ratings clearly indicated an increase in negative and decrease in positive affective state throughout the procedure. A higher concentration in hair cortisol, which reflects a cumulative stress parameter over the course of the last 2–3 months, was associated with a stronger drop in (most pronounced) positive PANAS ratings over the course of the biopsy. These findings suggest that subjects with higher stress levels prior to the biopsy were more likely to develop negative/distressed mood throughout the procedure. Likewise a higher depression score, assessed outside the scanner, had a more negative impact on the mood during the biopsy suggesting that a screening prior to biopsy could help identifying people at risk for developing more distress by diagnostic procedures.

Higher well-being and emotional stability has been linked to enhanced emotion regulation abilities^17^. A recent meta-analysis on the effectiveness of emotion regulation strategies in older versus younger adults^18^ found no major differences in the effectiveness of reappraisal or distancing strategies and only mildly stronger effects for response modulation techniques in older adults. Potentially related to this, our findings indicate that a higher tendency towards suppression of emotional facial expressions and feelings in general, as indicated by the emotion regulation questionnaire is associated to a weaker drop in positive affect over the course of the biopsy, but also to an increase in negative affect. These finding are in line with the notion that older adults might be more efficient in selecting and deploying regulation strategies as opposed to being more efficient in the strategies themselves^17–19^. The increased within network functional connectivity in experienced meditators^20^ and social regulation experts^21^, with increased automaticity of a cognitive process^22^ and with increasing age^23^. Thus, the influence of within default mode network connectivity on the change of positive and negative affect might reflect this habitual emotion regulation use. As a cautionary note, the aging brain shows sex-specific changes in stress responsivity, indicating that any generalization to women should be done with caution^24^.

Our data confirmed the significant impact of a real-life stressor in large-scale brain networks that have previously been associated with stress regulation^9,10,15,16^. We found evidence for a strong within SAL connectivity and an elevated connectivity with the other two networks. This finding is in line with an upregulation of salience network when individuals are acutely stressed and with other findings demonstrating an elevated connectivity of the salience network and the DMN after social stress induction^15^. The salience network has been found to show elevated within network connectivity in PTSD^25,26^, which decreases after successful treatment^27^. That result may point to a chronic dysregulation of an acute stress response in PTSD. Our results indicate that MRI based prostate biopsy is perceived as an acute stressor, which is associated with typical large-scale brain network reconfigurations of an acute stress response. A potential clinical implication of our findings is that identifying stress sensitivity and preventing stress in sensitive individuals is relevant in larger interventions and for example for other forms of outcome such as pain medication. Moreover, our results clearly show that these invasive diagnostics in the elderly are stressful and need to be thoroughly accommodated by professionals. A limitation one needs to keep in mind is that these real-life experiments for ethical reasons do not allow collecting data on a sham control or alternative placebo procedures, which could partially explain differences in subject responses.

In sum, the current findings are relevant to understand the impact of an acute real life stressor as well as the impact of diagnostic interventions whereby we are of course aware of the shortcoming with respect to lack in demographical diversity and sample size. Our proof of concept data suggests that diagnostic procedures performed with a potentially threatening cancer diagnosis lead to extra distress. Many studies have investigated the impact of such an environmental stressor in relation to coping strategies. Here we include the relationship between brain and behavior and can demonstrate that the interaction between the different resting state networks may be associated with or even predict the behavioral response. We suggest considering that an aging population undergoing diagnostics for potentially negatively impacting diseases might have limited options of managing pain and distress. It has been noted that non-pharmacologic approaches in these settings are largely under-investigated and initial behavioral studies by Lang and colleagues are promising with respect to stress reduction during MRI procedures^2,28^. Future studies may compare the impact of the different procedures but it is evenly important to include the individual level of vulnerability as indexed by behavioral screening prior to the intervention.

## Material and methods

We conducted a prospective, non-randomized, single center proof of concept study at the Radboud University Medical Centre, Nijmegen, Netherlands.

All patients were referred to the department of radiology for further MR -guided biopsy since they had an elevated PSA level or abnormal digital rectal examination and a suspicious finding (PIRADS 3 to 5) on prostate MRI examination. Exclusion criteria of the study were contra-indications to undergo MRI, impossibility to obtain a valid informed consent, history of psychiatric treatment or current psychiatric treatment as revealed by self-report, history of neurological treatment or current neurological treatment as revealed by self-report, history of endocrine treatment or current endocrine treatment as revealed by self-report. The study was conducted according to the in accordance with the Declaration of Helsinki in its latest form^29^ approved by the CMO Regio Arnhem-Nijmegen (Dosiernummer: 2015-2182; NL-Nummer: NL55573.091.15) and all participants signed an informed consent prior to participation in the study.

### Procedure

Before the start of the MRI-guided biopsy procedure, patients were asked to fill in a questionnaire on their subjective affective state (Positive and Negative Affect Scale; PANAS^30^; see Fig. 1) and a saliva sample for measurement of cortisol levels was obtained using Salivette collection devices (Sarstedt, Rommelsdorf, Germany). Next, patients underwent brain imaging for which they lay in a supine position on the same MR table with their head in the middle of the bore in a “bird-cage” coil. After the additional brain scan, a second PANAS measurement and saliva sample was acquired and patients were repositioned for the MRI-guided biopsy in a prone position. After biopsy, patients filled in another PANAS and gave another saliva sample. Additionally, patients were asked to fill in the PANAS and collect one additional sample (baseline) on the same time-of-day 1 week after the visit. All saliva samples were stored at − 20 °C until assaying. Biochemical analysis of free cortisol in saliva is performed using a competitive electrochemiluminescence immunoassay (ECLIA, Elecsys 2010, Roche Diagnostics). Due to technical problems during analyses, contamination of the samples, no saliva in the tubes and samples being lost in the mail delivery we lost data from a total of 8 samples from 7 individuals. Prior to this procedure and after 1 week back at home, patients filled in the Depression Anxiety Stress Scales 21 (DASS-21)^31,32^, the Coping Inventory for Stressful Situations-21 (CISS-21^33^ the Beck Depression Inventory-II (BDI^34,35^) the Emotion Regulation Questionnaire (ERQ^36^) and the Childhood Trauma Questionnaire (CTQ-SF^37^).

### Imaging sequence and analyses

MRI imaging was conducted at a 3 T scanner (Skyra, Siemens Healthcare, Erlangen, Germany) at the Radboud University Medical Center. A 3D magnetization-prepared rapid gradient echo (MPRAGE) anatomical T1-weighted image was acquired for normalization purposes (192 slices, 256 × 256 matrix, FoV of 240 × 240 mm^2^ with isotropic 0.9 mm^3^ voxels, TR = 2300 ms, TE = 2.32 ms; α = 8°). Next a resting state scan was done for 6 min using echo-planar imaging sensitive to BOLD contrast (T2*, voxel size: 3 × 3 × 3 mm^3^, 64 × 64 matrix, FoV: 195 mm^2^, 44 slices, gap 0 mm, TR 2.5 s, TE 30 ms, α = 90°; repetitions = 144; interleaved acquisition).

### Preprocessing of fMRI data

MR data quality checks were performed by visual inspection of the structural and functional scans, spike checks and signal-to-noise (SNR) ratio plots. FSL (FMRIB, University of Oxford, UK; http://www.fmrib.ox.ac.uk/fsl^38^ was used for pre-processing, data-denoising, group analyses and assessment of network connectivity strength. For pre-processing, the first 5 volumes of each functional time series were discarded, allowing magnetization effects and initial transient signal changes. Further pre-processing steps included three-dimensional movement correction, and spatial smoothing using a 5 mm full-width at half maximum (FWHM) Gaussian kernel to reduce inter-subject variability and a high-pass filter (> 0.007 Hz) was applied. All pre-processing steps except temporal filtering were conducted before AROMA data denoising^39,40^. Briefly, ICA-AROMA is designed to identify motion related artifacts by matching single subject ICA components to four robust and standardized features. The data is denoised by linear regression of ICA components identified as noise by AROMA and subsequently the high pass filter was applied. Prior to all group analyses data were normalized to MNI space and re-sampled to 2 mm^3^ resolution using FMRIB’s Nonlinear Image Registration Tool (FNIRT).

We focused on connectivity strength in three large-scale brain networks that are known to be modulated during an adaptive stress response: the executive-control network (ECN), the salience network (SAL) and the default mode network (DMN)^9,10,16^. Initially, we created three masks for the respective networks based on previous work^9,41^. These three masks were constructed based on a combination of functional templates and anatomical atlases. For the default mode network, the midline core hubs^41^ and the parietal hubs were extracted from the Smith template^6^, thresholded at an arbitrary threshold of z > 5 and binarized. For the salience and executive control networks we took a theory based approach, as data-driven decompositions do not overlap with conceptual thinking of salience and executive control network with regard to topoplogy. We based the selection of core hubs on Hermans et al.^9^. For the salience network we took the cingulate mask from the Harvard–Oxford atlas and drew the border for the anterior cingulate manually based on Vogt’s differentiation of the subregions of the cingulate cortex^42^. Similarly, the anterior insula was taken from Harvard–Oxford and isolated from the posterior based on topological knowledge^43–45^. For the ECN, the paracingulate gyrus was taken from Harvard–Oxford and segregated at the same border as the cingulate gyrus. Furthermore, the middle frontal gyrus and the supramarginal gyrus were extracted from Harvard–Oxford as best matches to the functional hubs denoted in Hermans and colleagues. Masks can be optained from https://neurovault.org/collections/MFDMBANR/). All data will be made available upon publication in the Donders Sharing Repository (10.34973/cesj-dk54).

We used the dual (spatial and temporal) regression technique to generate subject-specific functional connectivity strength values per network^46^. For this, we calculated the median for each network mask in the three statistical maps that were identified during the in the dual regression. These maps contain subject and voxel-wise estimates of connectivity strength for the three networks. Thus, we generated three estimates of within network connectivity strength per subject.

### Data analysis

To analyze changes of physiological (salivary cortisol) and psychological stress (PANAS self ratings), we first calculated baseline corrected scores for salivary cortisol and PANAS values by dividing all scores taken during the biopsy procedure by the baseline measurement once week after biopsy. This procedure controls for between subject differences in salivary levels and subjective affect. The baseline corrected values were entered into repeated measures ANOVAs. Cortisol values were analyzed for time effects over the three repeated measurements. In the analysis of PANAS values, the factor of valence was added, as the PANAS consists of positive and negative affect subscales. In both analyses, hair cortisol and within-network functional connectivity of the three networks of interest were added as covariate. Greenhouse–Geisser corrected values are reported due to violation of sphericity.

#### Prediction of subjective and physiological stress

In order to evaluate the interaction of adaptive stress responses during the biopsy procedure with trait measures human behavior, physiological trait stress markers (hair cortisol) and brain connectivity dynamics related to stress we conducted two separate explorative regression analysis for physiological stress responses (cortisol) and subjective affective stress responses (PANAS). For both we took the baseline corrected values and subtracted the last from the first measurement, to acquire one value indicating change under the biopsy procedure. We included anxiety and depression scores from the DASS 21, the summative depression score from the BDI, emotion regulation styles (reappraisal, suppression) from the ERQ, hair cortisol and the three indices of within network connectivity strength from the ECN, SAL and DMN. We ran a step-wise regression with inclusion of predictor variables at p < 0.05 and at each step an exclusion criterion of p > 0.1.

## Supplementary Information

Supplementary Video Legends.

Supplementary Video S1.

Supplementary Video S2.

Supplementary Video S3.

Supplementary Video S4.

Supplementary Video S5.

Supplementary Video S6.

## Data Availability

Masks and results images can be optained from https://neurovault.org/collections/MFDMBANR/. All data will be made available in the Donders Sharing Repository upon publication.
