# Supplementary material for: Measuring inter-individual differences in stress sensitivity during MR-guided prostate biopsy
Source: Sci Rep. 2021 Jan 28;11:2454. doi: 10.1038/s41598-021-82199-z (PMC7844251; doi:10.1038/s41598-021-82199-z)
Supplement: Supplementary file 1 — Supplementary Video Legends. [file 41598_2021_82199_MOESM1_ESM.docx]

Supplementary Material for:

Measuring inter-individual differences in stress sensitivity during MR-guided prostate biopsy

Running title: Stress and prostate biopsy

Nils Kohn^1*^, Jan Heidkamp^2^, Guillén Fernández^1^, Jurgen Fütterer^2^, Indira Tendolkar^3^

^1^Donders Institute for Brain Cognition and Behavior, Department of Cognitive Neuroscience, Radboud University Medical Center, Nijmegen, The Netherlands

^2^Department of Medical Imaging, Radboud University Medical Center, Nijmegen, The Netherlands

^3^Donders Institute for Brain Cognition and Behavior, Department of Psychiatry, Radboud University Medical Center, Nijmegen, The Netherlands

* corresponding author: Nils Kohn; email: [nils.kohn@gmx.net](mailto:nils.kohn@gmx.net)

List of files:

SupplementaryVideo1_DMNmask.avi – a video of the standard MNI brain rendered mask for DMN

SupplementaryVideo2_ECNmask.avi - a video of the standard MNI brain rendered mask for ECN

SupplementaryVideo3_SALmask.avi - a video of the standard MNI brain rendered mask for SAL

SupplementaryVideo4_DR_DMN.avi - a video of the standard MNI brain rendered group results for the dual regression of the DMN

SupplementaryVideo5_DR_ECN.avi - a video of the standard MNI brain rendered group results for the dual regression of the ECN

SupplementaryVideo6_DR_SAL.avi - a video of the standard MNI brain rendered group results for the dual regression of the SAL
